# Supplementary material for: The causal relationship between the human gut microbiota and pyogenic arthritis: a Mendelian randomization study
Source: Front Cell Infect Microbiol. 2024 Nov 26;14:1452480. doi: 10.3389/fcimb.2024.1452480 (PMC11629706; doi:10.3389/fcimb.2024.1452480)
Supplement: Supplementary file 1 [file DataSheet1.zip › mendelian test/ebi-a-GCST90027578.csv_forest.pdf]

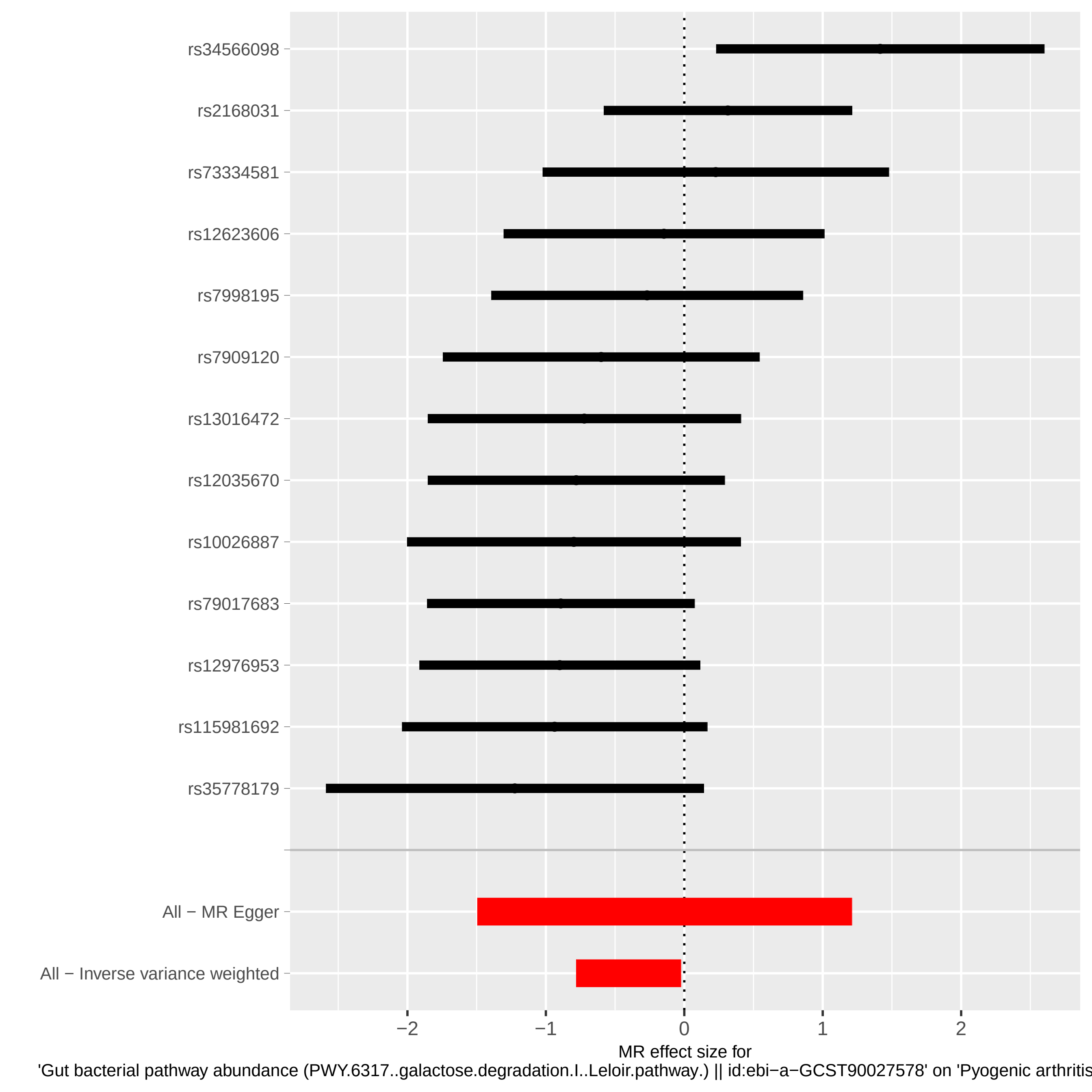

rs34566098
rs2168031
rs73334581
rs12623606
rs7998195
rs7909120
rs13016472
rs12035670
rs10026887
rs79017683
rs12976953
rs115981692
rs35778179
All − MR Egger
All − Inverse variance weighted
−2
−1
0
1
2
MR effect size for
'Gut bacterial pathway abundance (PWY.6317..galactose.degradation.I..Leloir.pathway.) || id:ebi−a−GCST90027578' on 'Pyogenic arthritis
